# Incremental value of radiomics with machine learning to the existing prognostic models for predicting outcome in renal cell carcinoma

**DOI:** 10.3389/fonc.2023.1036734

**Published:** 2023-04-28

**Authors:** Jiajun Xing, Yiyang Liu, Zhongyuan Wang, Aiming Xu, Shifeng Su, Sipeng Shen, Zengjun Wang

**Affiliations:** ^1^ Department of Urology, The First Affiliated Hospital of Nanjing Medical University, Nanjing, China; ^2^ Department of Biostatistics, School of Public Health, Nanjing Medical University, Nanjing, Jiangsu, China

**Keywords:** renal cell carcinoma, radiomics, prognostic model, machine learning, computed tomography running title, incremental value estimation

## Abstract

**Purpose:**

To systematically evaluate the potential of radiomics coupled with machine-learning algorithms to improve the predictive power for overall survival (OS) of renal cell carcinoma (RCC).

**Methods:**

A total of 689 RCC patients (281 in the training cohort, 225 in the validation cohort 1 and 183 in the validation cohort 2) who underwent preoperative contrast-enhanced CT and surgical treatment were recruited from three independent databases and one institution. 851 radiomics features were screened using machine-learning algorithm, including Random Forest and Lasso-COX Regression, to establish radiomics signature. The clinical and radiomics nomogram were built by multivariate COX regression. The models were further assessed by Time-dependent receiver operator characteristic, concordance index, calibration curve, clinical impact curve and decision curve analysis.

**Result:**

The radiomics signature comprised 11 prognosis-related features and was significantly correlated with OS in the training and two validation cohorts (Hazard Ratios: 2.718 (2.246,3.291)). Based on radiomics signature, WHOISUP, SSIGN, TNM Stage and clinical score, the radiomics nomogram has been developed. Compared with the existing prognostic models, the AUCs of 5 years OS prediction of the radiomics nomogram were superior to the TNM, WHOISUP and SSIGN model in the training cohort (0.841 vs 0.734, 0.707, 0.644) and validation cohort2 (0.917 vs 0.707, 0.773, 0.771). Stratification analysis suggested that the sensitivity of some drugs and pathways in cancer were observed different for RCC patients with high-and low-radiomics scores.

**Conclusion:**

This study showed the application of contrast-enhanced CT-based radiomics in RCC patients, creating novel radiomics nomogram that could be used to predict OS. Radiomics provided incremental prognostic value to the existing models and significantly improved the predictive power. The radiomics nomogram might be helpful for clinicians to evaluate the benefit of surgery or adjuvant therapy and make individualized therapeutic regimens for patients with renal cell carcinoma.

## 1 Introduction

The most common malignant tumor in the kidney is renal cell carcinoma (RCC), which originates from the proximal tubular epithelial system of the renal parenchyma, and more than 60,000 people worldwide suffer from it every year (1–3). According to European Association of Urology Guidelines, surgical treatment is the first choice for patients with RCC, of whom the overall 5-year survival rate was in the range of 50–60% (4, 5).

RCC is recognized as having a highly variable natural history, according to the previous reports (6). RCC patients have different responses to surgical treatment and prognosis (7). Many prognostic models for RCC have previously been developed to provide prognostic assessment for patients and to inform clinical management strategies and improve risk stratification for clinical trials, including prognostic scores based on TNM stage, tumor size, nuclear grade and necrosis (SSIGN), tumor-node-metastasis (TNM) stage and WHOISUP (8). According to a report from a retrospective study evaluating 358 patients with RCC, the predictive efficiency of the SSIGN model was slightly better than that of the TNM stage system (9).

According to the European Association of Urology guidelines, among patients with stage I-II localized RCC, neoadjuvant therapy is still experimental, and chemotherapy and targeted therapy are not standard treatments for most patients (10). Especially for resectable tumors, it should not be routinely presented outside of clinical trials. However, despite following postoperative surveillance guidelines, approximately 20% to 30% of patients with TNM stage I and II RCC who were considered to have a better prognosis would develop recurrence or metastasis after surgery (11, 12). Therefore, existing prognostic models showed certain limitations in current clinical practice, and the ability to accurately predict individual patient outcomes remained limited. An accurate and simple RCC prognostic tool is still urgently needed.

Tumor heterogeneity is defined as tumor cell with distinct molecular and phenotypic characteristics. Recent evidence suggested that the level of tumor heterogeneity could serve as a prognostic biomarker (13). Tumor heterogeneity manifests at multiple spatial dimensions, mainly including genetic, cellular, histological, and radiological levels. The TNM stage and SSIGN score system are mainly based on the anatomical and histological features of tumors, which cannot reflect the heterogeneity of tumors and may not be sufficient to provide accurate prognostic information for RCC patients (14, 15). In current clinical practice, the phenotypic heterogeneity of RCC was mainly assessed by biopsy-based microscopy and gene expression analysis. However, the capabilities of genomics, proteomics or histology were limited. It was difficult to assess intratumor heterogeneity well with a random sample alone.

Radiomics is a promising approach to automatically mine a lot of quantitative image features that are difficult to identify with the naked eye and reveal aspects of intratumor heterogeneity with potential prognostic relevance (16, 17). The application of machine learning in radiomics has emerged as a non-invasive and low-cost method for accurate prognosis assessment.

The aim of this study was to identify radiomics features associated with overall survival in RCC, to evaluate its incremental value to clinical characteristics and other existing prognostic models, to establish a visual nomogram for patients with RCC and to provide reference for neoadjuvant therapy and surgical plan.

## 2 Related work

Radiomics refers to the extraction of high-throughput quantitative features from radiographic images, the in-depth non-invasive analysis of tumor heterogeneity across the tumor volume, and the establishment of predictive models that correlate imaging features with genomic patterns and clinical outcomes. Recently, radiomics has been widely applied in tumor imaging-based diagnosis, prognosis prediction, and efficacy monitoring. Some of the studies were presented below:

According to previous reports, among patients with stage I lung adenocarcinoma, the radiomics signature was associated with overall survival. The clinical-radiomics nomogram could accurately predict Axillary lymph node metastasis (ALNM) (AUC: 0.92) (18). The model, integrating clinical variables and radiomics features, had good performance for predicting Microvascular invasion (MVI) and clinical outcomes (19). Meanwhile, Ruizhi Gao et al. provided a predictive nomogram that integrates radiomic and clinicopathological characteristics for predicting the progression-free interval (PFI) of kidney renal clear cell carcinoma patients (20). Mostafa Nazari et al. developed a robust radiomics-based classifier that was capable of accurately predicting overall survival of RCC patients for prognosis of ccRCC patients (21).

In addition, Multiparametric MRI (mpMRI) allows assessment of the anatomical and functional characteristics of the renal mass. Using diffusion MRI, parenchymal wash index, and ADC ratio were correlated with clear-cell RCC Fuhrman grade, with a pooled sensitivity and specificity of DWI to differentiate between high and low grades of 78% and 86%, respectively (22, 23).

Most of the above studies have used texture analysis, meanwhile, other studies have also used convolutional neural network (CNN). An ensemble model based on residual convolutional neural network (ResNet) was built combining clinical variables and T1C and T2WI MR images using a bagging classifier to predict renal tumor pathology. Stavropoulos et al. found that compared with all experts averaged, the ensemble deep learning model had higher test accuracy (0.70 vs. 0.60, P = 0.053), sensitivity (0.92 vs. 0.80, P = 0.017), and specificity (0.41 vs. 0.35, P = 0.450) (24).

## 3 Materials and methods

### 3.1 Study design and patients

A total of 689 patients with renal cell carcinoma confirmed by histology were recruited in this study. 281 RCC patients came from the First Affiliated Hospital of Nanjing Medical University (NJMU) from 2010 to 2019. In addition, 408 patients were collected from three external database (Clinical Proteomic Tumor Analysis Consortium (CPTAC), Kidney Tumor Segmentation Challenge (KITS) and The Cancer Imaging Archive (TCIA)). The criteria for inclusion and exclusion are as follows: i) patients with complete baseline and follow-up information; ii) patients with contrast-enhanced CT imaging including arterial phase before surgical resection; iii) patients histologically confirmed RCC; iv) no imaging artifacts. The detailed flowchart summarizing patient inclusion and exclusion in this study was presented in **Figure S1**.

In the phase of model development, we used the NJMU as the training cohort (N=281) and the remaining datasets (CPTAC, TCIA, and KITS) as the two independent validation cohorts (N1 = 225, N2 = 183). The validation cohort1 contained the patients in TCIA and CPTAC (NCI) datasets while the validation cohort2 contained the patients in KITS dataset. All relevant data was collected in July 2020, and for patients who could not visit the hospital, a follow-up phone call was conducted. The overall survival was calculated from the date of pathological diagnosis to the time of death or the last follow-up. Baseline data consisted of Age, Gender, Body Mass Index (BMI), TNM stage, WHOISUP, Tumor size, Laterality, Location, Tumor margin, SSIGN. The TNM stage is based on The Union for International Cancer Control tumor node metastasis staging system (8). In SSIGN, risk points are accumulated and added up to provide a risk score (25).

The primary tool to assess frailty was the modified frailty index of the Canadian Study of Health and Aging (11-CSHA), which is a validated tool based on clinical data and consisting of eleven elements. The sum score is divided by 11 and a cut-off of ≥ 0.27 has been defined to mirror frailty (26).

### 3.2 Radiomics feature extraction

In the training set, a total of two types of CT scanners are involved, including Philips iCT 256 (Koninklijke Philips, Nevada, USA) and Somatom force CT A50A (Siemens Healthcare GmbH, Erlangen, Germany). The detail CT scanning parameters are shown in **Table S1**.

**Figure 1:**
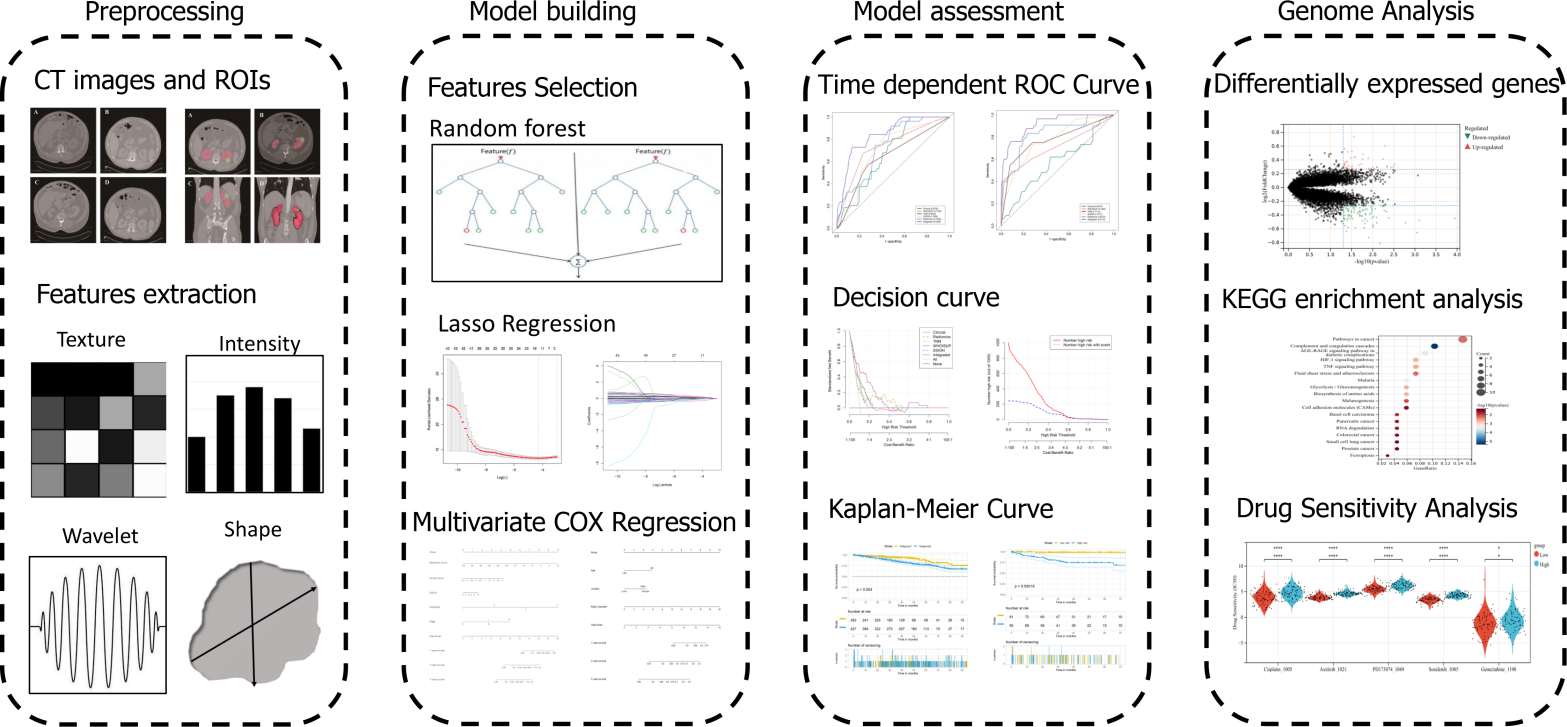
Graphical abstract of radiomics analysis and model building.

The workflow of the study was shown in **Figure 1**. The regions of interest (ROIs) in the RCC were separately manually slice-by-slice contoured and segmented using 3D Slicer (version 4.11.2) by two urologists (Yiyang Liu and Shifeng Su), who were not informed of the patients’ personal information. Each of them with at least 10 years of clinical experience in kidney CT, took full responsibility for the ROI delineation. The ROIs were evaluated by experts following the medical imaging standards. After the ROIs of the SRMs were delineated, the CT images were transferred to a radiomics plugin for 3D Slicer (PyRadiomics). Then, the extraction of in-house radiomics features was performed using PyRadiomics package. Each ROI in contrast-enhanced CT imaging (arterial phase) had eight sets of radiomics features. The voxel-based features included shape 2D, shape 3D, first-order, gray-level cooccurrence matrix (GLCM), gray-level size zone matrix (GLSZM), gray-level dependence matrix (GLDM), gray level run length matrix (GLRLM) and neighbouring gray tone difference matrix (NGTDM), containing a total of 851 quantitative features. The Bland-Altman test was used for assessment of interobserver variability.

The extracted radiomics features in each dataset were further normalized with mean value=0 and standard deviation=1 to make all the variables comparable across different dataset and applicable easily in the future.

### 3.3 Unsupervised clustering and subgroup discovery

Unsupervised hierarchical clustering, based on the raw radiomics data scaled by mean and centered, was performed using cutree package in R. Kaplan-Meier overall survival curves were plotted using survival package in R. The statistical difference in survival between the two patient subgroups was calculated with the coxph function. The heatmap was plotted using the pheatmap package in R.

### 3.4 Development of the radiomics signature

Random Forest was performed using the ranger package. The random forest feature importance was obtained from ranger-package’s variable-importance-parameter on a trained random forest model. In the training cohort, the radiomics features associated with overall survival were screened by the least absolute shrinkage and selection operator (LASSO) Cox regression. The Radiomics signature was calculated with a linear combination of the selected radiomics features multiplied by their corresponding LASSO-Cox coefficients. Based on median radiomics score, patients were classified as high-risk or low-risk group. Kaplan-Meier overall survival analysis was performed between the stratified subgroups.

### 3.5 Development of the radiomics nomogram

The association between clinical characteristics and overall survival was separately evaluated by the univariable and multivariable Cox regression analysis. The hazard ratio (HR) of each predictor was obtained simultaneously. The clinical nomogram and radiomics nomogram, which predicted 1-, 3- and 5-year overall survival, were separately constructed using the rms and survival package in R. Based on TMN Stage, WHOISUP and SSIGN, the prognosis model for OS Prediction has been developed by survival package in R.

### 3.6 Identification of DEGs and functional enrichment analysis

The differentially expressed genes (DEGs) were identified between the high- and low-risk subgroups using the limma package with criteria of |log2-fold change (FC)| ≥ 1 and p-value < 0.05.

The KEGG enrichment analysis was performed using the clusterProfiler package to obtain the results of gene set enrichment. For Gene set enrichment analysis (GSEA), the GSEA software was obtained from the GSEA website (http://software.broadinstitute.org/gsea/index.jsp) to evaluate related pathways and molecular mechanisms.

### 3.7 Mutation and drug sensitivity analysis

The mutational profiles of RCC patients between high and low risk subgroups were identified using the maftools package. To explore the sensitivity of antineoplastic drugs in RCC patients, the semi-inhibitory concentration (IC50) values of common drugs was calculated using the oncoPredict package.

### 3.8 Statistical analysis

Statistical tests were performed with R statistical software. To evaluate the performance of the prognosis model, we used calibration curves constructed by rms package. The performance of the models was evaluated by the time-dependent area under the curve (AUC) of receiver operator characteristic (ROC). The decision curve analysis (DCA) was performed with the rmda package. For all analyses, P < 0.05 was considered statistically significant.

## 4 Result

### 4.1 Basic characteristics

**Table S2** showed the clinical characteristics of the entire cohort (689 participants, median 58.8 years), training cohort (NJMU, 281 participants, median 57 years), validation cohort 1 (NCI, 225 participants, median 61 years) and validation cohort 2 (183 participants, median 58 years). The mean follow-up for patients in the entire cohort was 41 months; and the 5-year OS rates were 86.9%, which was slightly higher than 71% reported in localized RCC in the literature, which might well be attributable to loss of some patients without surgery.

### 4.2 Overview of radiomics profile in RCC

**Figure 2:**
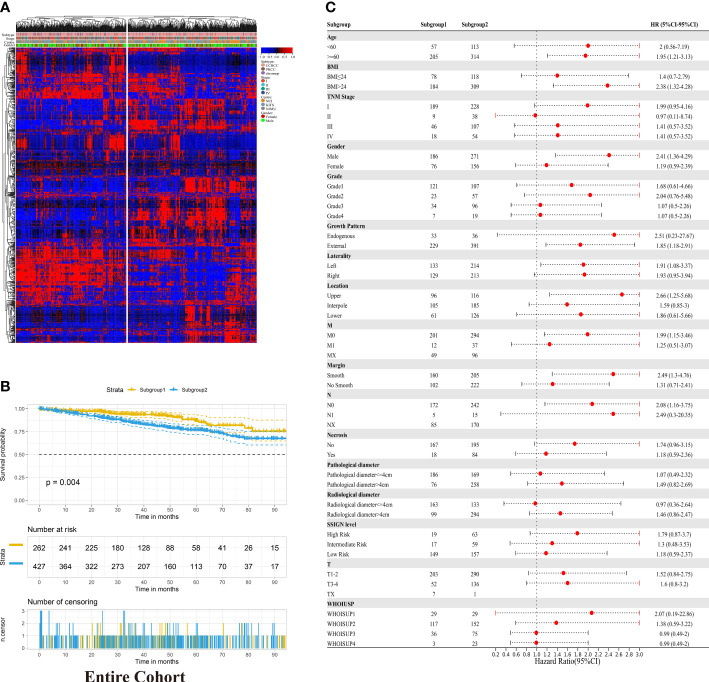
Unsupervised clustering analysis of radiomic data in RCC. **(A)** Unsupervised hierarchical clustering of radiomic profile from RCC identified two distinct subgroups. The associations between radiomic subgroups with gender, subtype and TNM stage are indicated on the right. **(B)** Kaplan-Meier analysis of the radiomic subgroups with OS in the entire cohort. **(C)** Hazard Ratios for radiomics grouping in each clinicopathological subgroup in the entire cohort.

To understand the radiomics features of RCC, the unsupervised hierarchical clustering analysis was conducted in the entire cohort. Based purely on the radiomics data, two distinct subgroups within RCC patients were identified (**Figure 2A**). Subgroup 2 was significantly associated with poor OS (p =0.004, log-rank test; **Figure 2B**). Furthermore, in the forest plot of the entire cohort, the significant Hazard Ratios were found for the subgroup, more specifically, in each age level (<60 and >=60), each BMI level (<=24 and >24), and regardless of TMN Stage, SSIGN level and WHOISUP (**Figure 2C**). In aggregate, unsupervised analysis suggested an intrinsic association between radiomics features and clinical characteristics, warranting further research.

### 4.3 Radiomics Signature for OS prediction with machine learning

**Figure 3:**
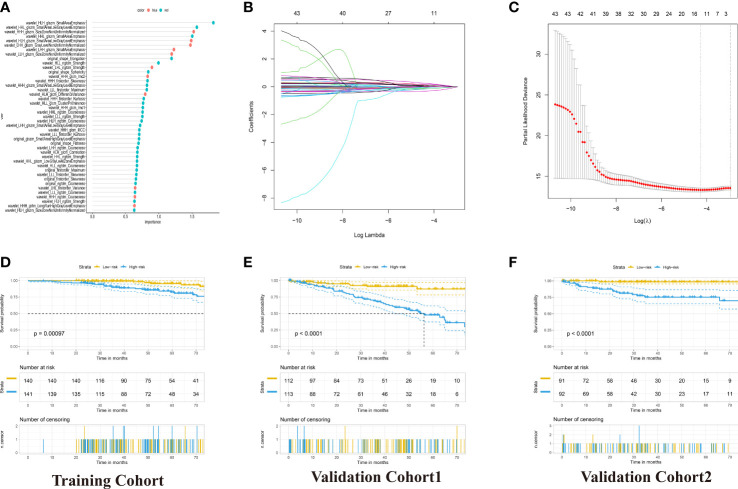
Development of the Radiomics Signature for OS Prediction with Machine Learning. **(A)** 43 radiomics feature importance from random-forest models. **(B)** The 5-fold cross-validation for tuning parameter screening in the LASSO regression model. **(C)** LASSO coefficient profiles of the features at different lambda values. **(D–F)** Kaplan–Meier curves for patients with High- and Low-Radiomics Score in the training and two validation cohorts.

To construct the radiomics signature, we extracted the 851 radiomics features of contrast-enhanced CT images. These features were screened in the random forest model to obtain the robust predictive factors. Based on the importance scores of features from the random forest model, the top five percent of the variables, 43 radiomics features were selected (**Figure 3A**; **Table S3**). Then, by LASSO-Cox regression analysis, 11 potential predictors from the 43 candidate variables were selected in the training cohort (**Figures 3B, C**). The calculation formula of radiomics signature was shown in the **Table S4**. Accordingly, the patients with RCC were divided into low-risk and high-risk groups. The high-risk group was significantly associated with poor OS in the three cohorts (p <0.001, log-rank test; **Figures 3D–F**).

**Figure 4:**
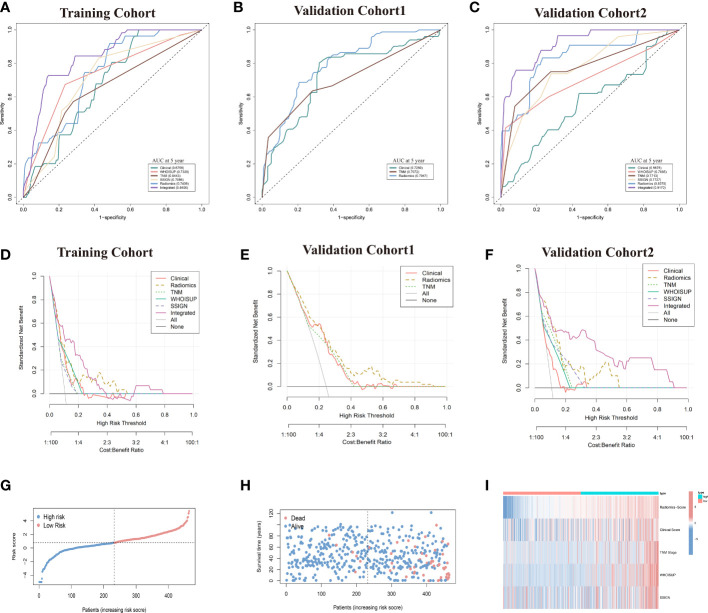
Prediction performance of the six models in the training and validation cohorts. **(A–C)** Time-independent ROC curves comparing the predictive accuracy of six models in the training and two validation cohorts. **(D–F)** Decision curves comparing six models among a series of risk thresholds in the training and two validation cohorts. **(G, H)** Ranked dot and scatter plots showing the radiomics-nomogram score distribution and patient survival status. **(I)** Component patterns of 5 selected prognostic factors in high- and low-risk groups for radiomics nomogram.

The prognostic power of the radiomics signature was assessed using time-dependent ROC analysis in the training cohort and two validation cohorts (**Figures 4A–C**). The radiomics model resulted in AUCs of 5-year OS prediction, 0.7409, 0.7947 and 0.830, respectively.

### 4.4 TNM stage, WHOISUP and SSIGN for OS prediction

The TNM Stage, WHOISUP and SSIGN are relatively common prognostic predictors. We put the three prognostic factors separately into the COX regression to establish overall survival prediction model. The above three prognostic predictors were negatively correlated to OS (HR for TNM Stage: 2.563, 95% CI: 1.246-5.271; HR for WHOISUP: 5.096, 95% CI: 2.435-10.670; HR for SSIGN: 1.2, 95% CI: 1.058-1.360).

The TNM Stage, WHOISUP and SSIGN models resulted in AUCs of 5 years OS prediction, 0.7409,0.7947 and 0.830, respectively, in the training cohort (**Figure 4A**) and 0.771,0.707 and 0.773 in validation cohort 2 (**Figure 4C**).

### 4.5 Clinical nomogram and radiomics nomogram for OS prediction

**Figure 5:**
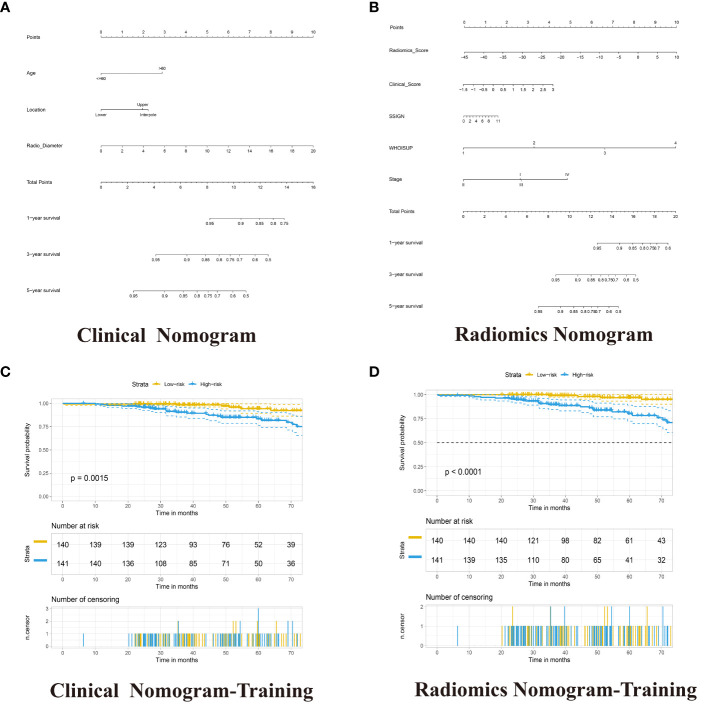
Development of the Clinical Nomogram and Radiomics Nomogram. **(A)** Nomogram for predicting the ratio of RCC patients with a certain survival time incorporating Age-level, Location and Radio-diameter in the training cohort. **(B)** Nomogram for predicting the ratio of RCC patients with a certain survival time incorporating Clinical score, SSIGN, TNM Stage, WHOISUP and radiomics signature in the training cohort. **(C, D)** Kaplan–Meier analysis of overall survival curves of High- and Low- Clinical or Radiomics nomogram in training group.

The results of univariate and multivariable COX regression analyses in the training cohort were shown in **Table S5**. The age-level, location and radio-diameter were significantly associated with OS, and these factors as independent prognostic factors were used to develop the clinical nomogram (**Table S6**). The clinical nomogram could distinguish high-risk from low-risk patients in the training cohort (p =0.0015, log-rank test) and the validation cohorts (**Figures 5A, C**; **S2D, S2I**).

The radiomics nomogram was built with Radiomics signature, TNM Stage, WHOISUP, SSIGN and Clinical score by the multivariate Cox regression analysis (**Figure 5B**, **Table S7**). The radiomics nomogram could distinguish high-risk from low-risk patients in the training cohort (p<0.0001, log-rank test, **Figure 5D**) and the validation cohort1 (p<0.001, **Figure S2H**). The clinical nomogram and radiomics nomogram resulted in AUCs of 5 years OS prediction, 0.676 and 0.841 in the training cohort, respectively, and 0.567 and 0.917 in validation cohort 2 (**Figures 4A, C**).

### 4.6 Prediction performance of the models

The prediction performances of the six models (clinical nomogram, radiomics nomogram, TNM Stage model, WHOISUP model, SSIGN model and radiomics signature) were presented in **Table 1**.

**Table 1:** Performance of six prognostic models in training and validation cohorts.

Cohort	Training Cohort		Validation Cohort1		Validation Cohort2		Threshold
	AUC at 5 years	C index	AUC at 5 years	C index	AUC at 5 years		
Radiomics Signature	0.741	0.614(0.498,0.73)	0.795	0.707(0.625,0.789)	0.837	0.828(0.748,0.908)	0.275
Clinical Nomogram	0.676	0.709(0.627,0.791)	0.729	0.691(0.622,0.76)	0.567	0.719(0.617,0.821)	-0.193
WHOISUP Model	0.734	0.73(0.63,0.83)	–	–	0.707	0.791(0.681,0.901)	1.87
SSIGN Model	0.707	0.675(0.575,0.775)	–	–	0.773	0.74(0.617,0.863)	-0.183
TNM Stage Model	0.644	0.62(0.52,0.72)	0.707	0.741(0.665,0.817)	0.771	0.761(0.632,0.89)	0.9
Radiomics Nomogram	0.841	0.834(0.779,0.889)	–	–	0.917	0.923(0.878,0.968)	0.78

Compared with the existing prognostic models, the AUC of 5 years OS prediction of the radiomics nomogram was superior to the TNM, WHOISUP and SSIGN model in the training cohort (0.841 vs 0.734, 0.707, 0.644) and validation cohort 2 (0.917 vs 0.707, 0.773, 0.771). Radiomics provided incremental value to traditional models and improved the power to predict prognosis. The risk plot of radiomics nomogram suggested as risk score increased, overall survival time decreased and mortality rose (**Figures 4G, H**). Moreover, the heatmap of selected prognostic predictors was shown in **Figure 4I**.

**Figure 6:**
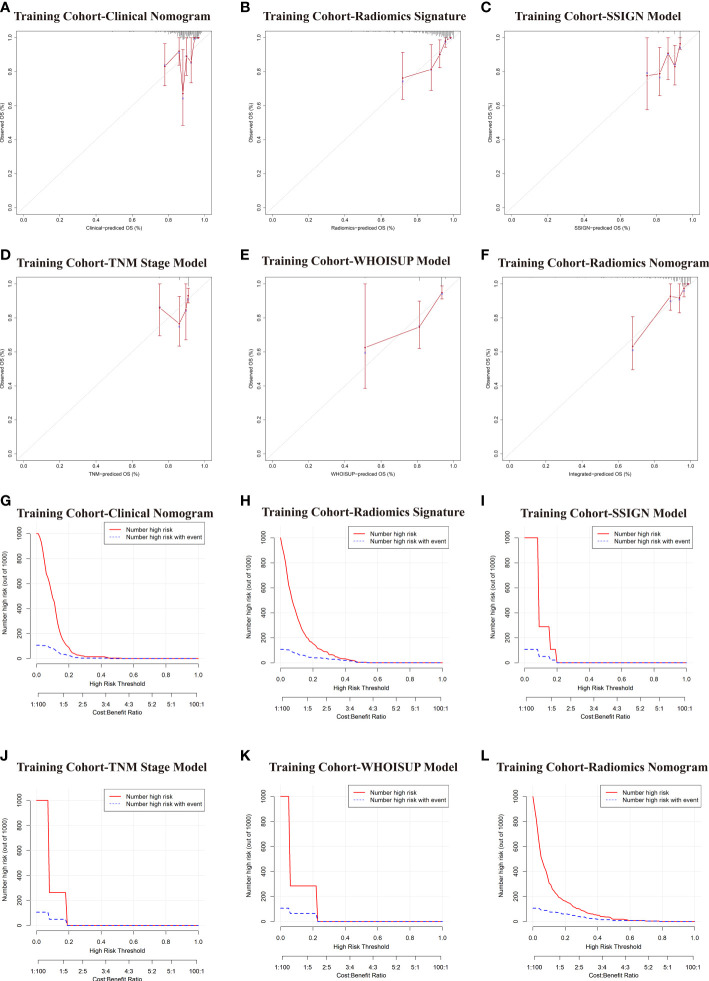
Comparative evaluation of various models in the training and validation cohorts. **(A–F)** Calibration plots describing the calibration of six models based on the consistency between predicted and observed 5-year OS results. **(G–L)** Clinical Impact Curve (CIC) of six models in the training cohort. The red curve (Number high Risk) represents the number of people classified as positive (high risk) by the model at each threshold probability; The blue curve (Number high risk with outcome) is the Number of true positives at each threshold probability.

The calibration curves of the radiomics nomogram for the probability of 5 years OS were presented in **Figure 6F**. The estimations with the radiomics nomogram were consistent with actual observations in the training, and 2 validation cohorts. And, the corresponding calibration curves of other models at 5 years were shown in **Figures 6**; **S3**.

To evaluate clinical applicability of these prognostic models, Clinical impact curve (CIC) analysis was conducted in **Figures 6G–L** and **Figure S3**. CIC visually indicated that the radiomics nomogram had a greater overall net benefit across a range of threshold probabilities, suggesting that the radiomics nomogram possessed significant prognostic value.

In the DCA analysis for the 6 prognostic models, the radiomics signature and radiomics nomogram showed superior overall net benefit over the existing prognostic models in predicting OS in the training and validation cohorts (**Figures 4D–F**). If the threshold probability of the traditional existing models was greater than 10% for predicting OS, radiomics nomogram had more benefit than either the SSISN, TNM Stage WHOISUP, or radiomics signature alone.

### 4.7 Biological interpretation and drug sensitivity of the radiomics signature

**Figure 7:**
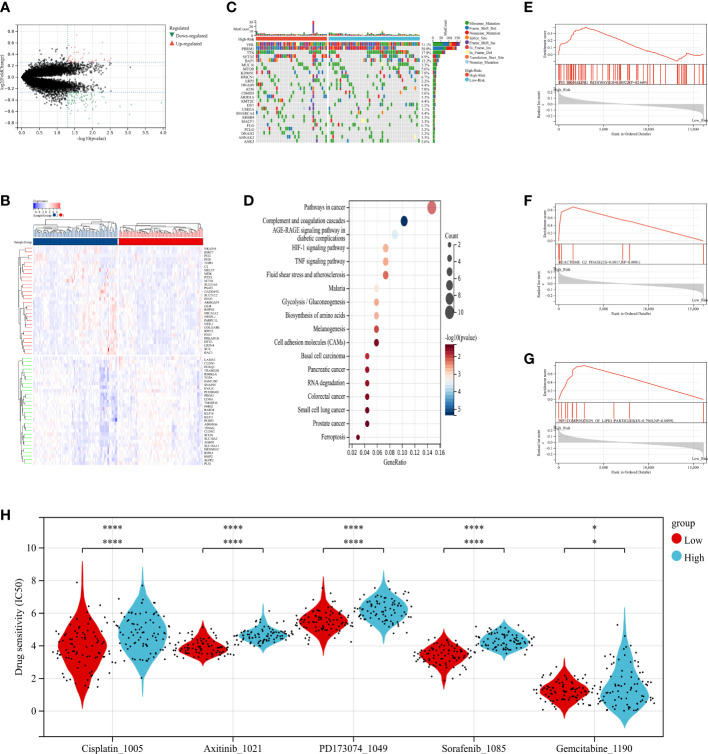
The relationship between radiomics and genomics in TCGA. **(A)** A volcano plot generated using the data of DEGs between high-risk and low-risk groups from TCGA. **(B)** The waterfall plot of somatic genetic alteration features established with high- and low- radiomics score. **(C)** Hierarchical clustering of 30 DEGs of RCC. **(D)** KEGG pathways analysis of DEGs associated with radiomics signature. **(E–G)** Gene Set Enrichment Analyses showed three representative pathways enriched in the high-radiomics score group. **(H)** Relationships between radiomics signature and drug sensitivity.

To explore the radiomics-related biological characteristics of renal cell carcinoma, based on their radiomics signatures, we divided the NCI validation cohort1 into high- and low-risk groups. We compared the transcriptome data of the two groups to find out the DEGs (**Figures 7A, C**; **S4A, S4C**). Then, we evaluated enrichments of KEGG pathways (**Figures 7D** and **S4B**); We found that HIF-1 signaling pathways and TNF signaling pathways were significantly enriched in the DEGs. Similarly, we identified that in gene set enrichment analysis (GSEA), for RCC patient samples in high-risk group, three signaling pathways were significantly enriched: P53-Signaling Pathway, G2 Phase and Composition of Lipid Particles (**Figures 7E–G**).

Additionally, the distribution differences of the somatic mutations were investigated between high-risk and low-risk groups in the TCGA-RCC dataset. As shown in **Figure 7B**, the VHL mutation incidence of were higher than 20% in RCC patients in two groups. Interestingly, compared with RCC in low-risk group, these tumor-related genes were more likely to be mutated in the high-risk group.

Further, to determine the power of radiomics signature to predict drug therapeutic response among RCC patients, the IC50 values of 198 drugs were evaluated in TCGA-RCC patients. We found that RCC patients in low-risk group might positively respond to Axitinib, Cisplatin, Gemcitabine, PD173074 and Sorafenib (**Figure 7H**). In summary, these findings suggested that radiomics signature was correlated with drug sensitivity.

### 4.8 Sensitivity analysis for the consistency of radiomics features

To evaluate the consistency and robustness of the extracted radiomics features, we further reanalyzed the CT images of our study using 3D Slicer by two urologists. The repeated radiomic features showed a high consistency (Pearson r, median (IQR): 0.71 (0.43-0.85)). 532 of 851 features (62.5%) had Pearson r > 0.6 (**Figure S5A**). Additionally, we estimated the mean difference between 11 duplicate radiomics features, and compared Bland-Altman plots of agreement between two observers (Bland and Altman, 1986) (**Figures S5B–L**; **Table S8**). There was high measurement agreement between two observers using the Bland-Altman test.

## 5 Discussion

In this study, we evaluated the predictive performance of the existing prognostic models for OS in RCC patients, and established a radiomics signature by machine learning algorithms and a radiomics nomogram by multivariate COX regression. We demonstrated that the addition of the radiomics signature significantly improved the predictive power of traditional prognostic models, the model’s ability to significantly classify patients into low- and high-risk groups, and its stability across both training and validation cohorts. Finally, our preliminary studies about genomics revealed a correlation between radiomics signature and the enrichment of certain pathways in tumors, as well as drug sensitivity.

The Tumor, Node and Metastasis (TNM) staging system is a method for stratifying cancer patients based on data from a large multicenter study involving a large number of patients and has a good level of evidence (15). TNM staging is also the most commonly used prognostic system for renal cell carcinoma. However, patients with the same TNM stage often could have various genetic and clinicopathological features and inconsistent survival rates. WHOISUP is also an important standard for the commonly used microscopic classification of renal cell carcinoma (27). According to the World Health Organization/International Society of Urological Pathology (WHO/ISUP) 4th edition grading system, RCC is classified into grades I to IV. Among RCC patients after surgery, including those with Partial nephrectomy (PN) and Radical nephrectomy (RN), the SSIGN score is a valuable prognostic tool (14). The existing prognostic models are widely recognized and used for post-operative management and clinical trial design. For patients with good or moderate prognosis, especially for patients with limited tumor burden and few symptoms, regular follow-up observation is generally recommended after surgery (28). Follow-up schedule for localized renal cell carcinoma after surgery should depend on the possibility of recurrence. CT scans of the chest and abdomen are routinely performed at intervals depending on the prognostic risk rating. For patients with poor prognosis, pembrolizumab combined with axitinib, lenvatinib and other drugs can be considered after surgery (29, 30). Therefore, accurate prediction of patient prognosis and risk stratification of RCC patients is an urgent problem.

It has been reported that frailty put patients undergoing surgery at a higher risk for developing poor healthcare outcomes (31). Besides tumor characteristics, patient characteristics, such as frailty, seemed to be the main aspects determining the postoperative outcome (32). However, the mean frailty score based on 11-CSHA score for patients was 0.18 ± 0.08 and 0.12 ± 0.06, respectively, in the training cohort and in validation cohort 2. There were only 16 patients (frailty score≥ 0.27) in the validation cohort2. This could be because frailty has been an important factor for the therapeutic strategy patients and clinicians choose as treatment for RCCs.

Although the above models took into account clinicopathological variables, tumor heterogeneity, which was thought to be associated with poorer patient outcomes, was not taken into account. It has been reported that the predictive power of these existing prognostic models might be significantly overestimated (33, 34). Although, tumor heterogeneity reduced the value of histopathology based on cell morphology and gene expression, it provided an opportunity for medical imaging to characterize whole tumors in a non-invasive and reproducible manner. In traditional radiology practice, images were typically evaluated visually or qualitatively, with the exception of a few measurements such as dimensions and volumes. This approach not only involved intra- and interobserver variability, but also left a large amount of deep hidden data in medical images that were not used, which limited the potential of precision medicine. In contrast, radiomics provides important complementary data on imaging phenotypes that may be informative (35).

Combining radiomic features, traditional staging systems, and other clinicopathological risk factors can improve the predictive power of tumor prognosis (36). In this study, a radiomics nomogram, combining radiomics signature with Clinical score, TNM stage, WHOISUP, and SSIGN prognostic factors, was established. The nomogram outperformed models using either radiomics or prognostic model alone (C index: 0.834 vs 0.62, 0.675, 0.73, 0.709). The radiomics nomogram combined multiple prognostic factors to accurately predict OS and stratify high and low-risk groups in RCC patients. This result was not surprising, as radiomics reflected higher-order imaging features that captured more tumor heterogeneity than macroscopic-level histopathological and clinical information. In this study, we included more patients with combined clinical characteristics and radiomics features to predict individualized survival with superior performance. Our discovery would take a critical step in which predictive models based on radiomics features could benefit physicians, patients and caregivers in managing RCC and facilitating personalized treatment.

Meanwhile, some studies have demonstrated different results due to the instability and low reproducibility of the radiomics model. A meta-analysis has indicated that image biomarkers based on the adjacent grey tone difference matrix and the size zone matrix should not be used in multi-center study, because these radiomics features were extremely sensitive to variations (37). Furthermore, CT images obtained from different hospitals may vary widely, thus leading to potential bias in multicenter studies. Multicenter normalization of medical images is the key to improve the predictive performance of radiomics-based applications. The robustness of reliability and repeatability is a major issue for clinical implementation of diagnosis and treatment prediction. In this study, the performance on both the training cohort and two independent validation cohorts was good (C index: 0.834 and 0.923), indicating that our prediction model was stable. Compared with previous studies that lacked interpretation of prognostic features, we comprehensively described biological and clinical characteristics associated with radiomics features that would help guide future clinical decision-making processes in a reliable and reproducible manner. Among the 11 selected radiomics features, GLCM, GLSZM and NGTDM measured the ROI array heterogeneity, with greater values of these features representing greater heterogeneity or a larger range of radiomics signature.

Additionally, in several previous studies, the prognostic models based on genomic and transcriptomic information of tumor tissues, such as gene expression, DNA methylation, CNAs, and non-coding RNAs were developed. It has been reported that a nomogram which was combined with six genes was able to accurately distinguish patients with higher risk of cancer-specific death (38). Meanwhile, Patrick et al. has established 13-gene signature whose expression levels could predict distinct outcomes of patients with RCC (39). But these molecular prediction models were difficult to translate into routine clinical applications because of the timeliness of tumor specimens and the large intratumor heterogeneity, resulting in insufficient prognostic power and high detection costs. The radiomics signature we proposed was simple and based solely on information from routine preoperative CECT scans at the time of patient onset. By observing the entire tumor area and extracting high-dimensional features such as wavelets and features, radiomics avoided tumor tissue features limited to a single site, and could mine more prognostic information than genomics. Therefore, it might serve as a surrogate biomarker for prognostic stratification of RCC patients.

And even more interesting, according to the radiomics hypothesis, intra-tumoral imaging heterogeneity might be an expression of underlying genetic heterogeneity that might lead to treatment resistance and thus suggested poorer prognosis. Based on DEGs between low-high radiomics score groups, some pathways in cancer, such as HIF-1 signaling pathways and TNF signaling pathways, were significantly enriched. The VHL regulated the drug sensitivity of renal cell carcinoma *via* HIF-1 pathway (40). The RNF26/CBX7 axis modulated the TNF pathway to promote Renal cell carcinoma proliferation (41). The RCC patients in low radiomics score group appeared to respond better to Axitinib, Cisplatin, Gemcitabine, PD173074 and Sorafenib, which have been approved for treatment of advanced renal cell carcinoma, than patients in high radiomics score group. Image data is a high-throughput macroscopic data. Based on macroscopic imaging data, we have found connections to the microscopic world, which may be a powerful tool for tumor prognosis and treatment prediction in the future.

Nevertheless, there were several limitations to the present study. First, in a retrospective study, bias was inevitable. Due to the retrospective nature of the study, the heterogeneity of abdominal enhanced CT versions and lack of algorithmic standardization existed across and within centers. Second, we did not assess the proportion of the patients died because of competing risks instead of progressive RCC. Third, the TCGA data alone could not prove that there was a difference between high- and low-radiomics score groups in tumor microenvironment and drug sensitivity. Fourth, RCC patients who did not undergo surgery for various reasons were not included in the study cohort. The absence of this group of patients might have caused selection bias in the study. Another limitation was the lack of information on the WHOISUP and SSIGN in validation cohort 1(NCI). Thus, to further validate these findings, a prospective multi-center study is needed.

In conclusion, we developed multiple prognostic models for RCC and evaluated their predictive performance, based on clinical characteristics and radiomics features from CECT. Radiomics signature provided statistically significant incremental value to the existing prognosis models in predicting OS and have broad clinical applications.

## Data availability statement

The raw data supporting the conclusions of this article will be made available by the authors, without undue reservation.

## Ethics statement

This study was approved by the Ethics Committee of the First Affiliated Hospital of Nanjing Medical University. The requirement for written informed consent was waived in view of the retrospective nature of the study (ethics approval number, 2021-SR-430).

## Author contributions

JJX, YYL and ZYW designed the research. JJX, YYL and SPS performed the data analysis and drafted the manuscript. ZYW and AMX collected clinical data. JJX, ZJW and SFS critically revised the manuscript. All authors contributed to the article and approved the submitted version. 

## Supplementary material

Supplementary Figure 1 Flow chart of the study population enrolment with inclusion and exclusion criteria.

Supplementary Figure 2 Kaplan–Meier curves for overall survival in the training and validation cohorts.

Supplementary Figure 3 Comparative evaluation of various models in the validation cohorts. **(A-J)** Calibration plots describing the calibration of six models based on the consistency between predicted and observed 5-year OS results. **(K-S)** Clinical Impact Curve (CIC) of six models in the validation cohort. The red curve (Number high Risk) represents the number of people classified as positive (high risk) by the model at each threshold probability; The blue curve (Number high risk with outcome) is the Number of true positives at each threshold probability.

Supplementary Figure 4 The relationship between radiomics and genomics in CPTAC. **(A)** Hierarchical clustering of 30 DEGs of RCC. **(B)** KEGG pathways analysis of DEGs associated with radiomics signature. **(C)** A volcano plot generated using the data of differentially expressed genes (DEGs) between high-risk and low-risk groups from CPTAC.

Supplementary Figure 5 Sensitivity analysis for the consistency of radiomics features. (A) Histogram of Pearson r values calculated from the radiomic features between the initial dataset and repeated dataset. (B-L) Bland-Altman plots were used to test the potential agreement between two observers. Mean difference (dashed line) is close to zero, showing no bias; solid black lines delimit limits of agreement (95% CI).
