# Supplementary figures and images for: Incremental value of radiomics with machine learning to the existing prognostic models for predicting outcome in renal cell carcinoma
Source: Front Oncol. 2023 Apr 28;13:1036734. doi: 10.3389/fonc.2023.1036734 (PMC10175776; doi:10.3389/fonc.2023.1036734)

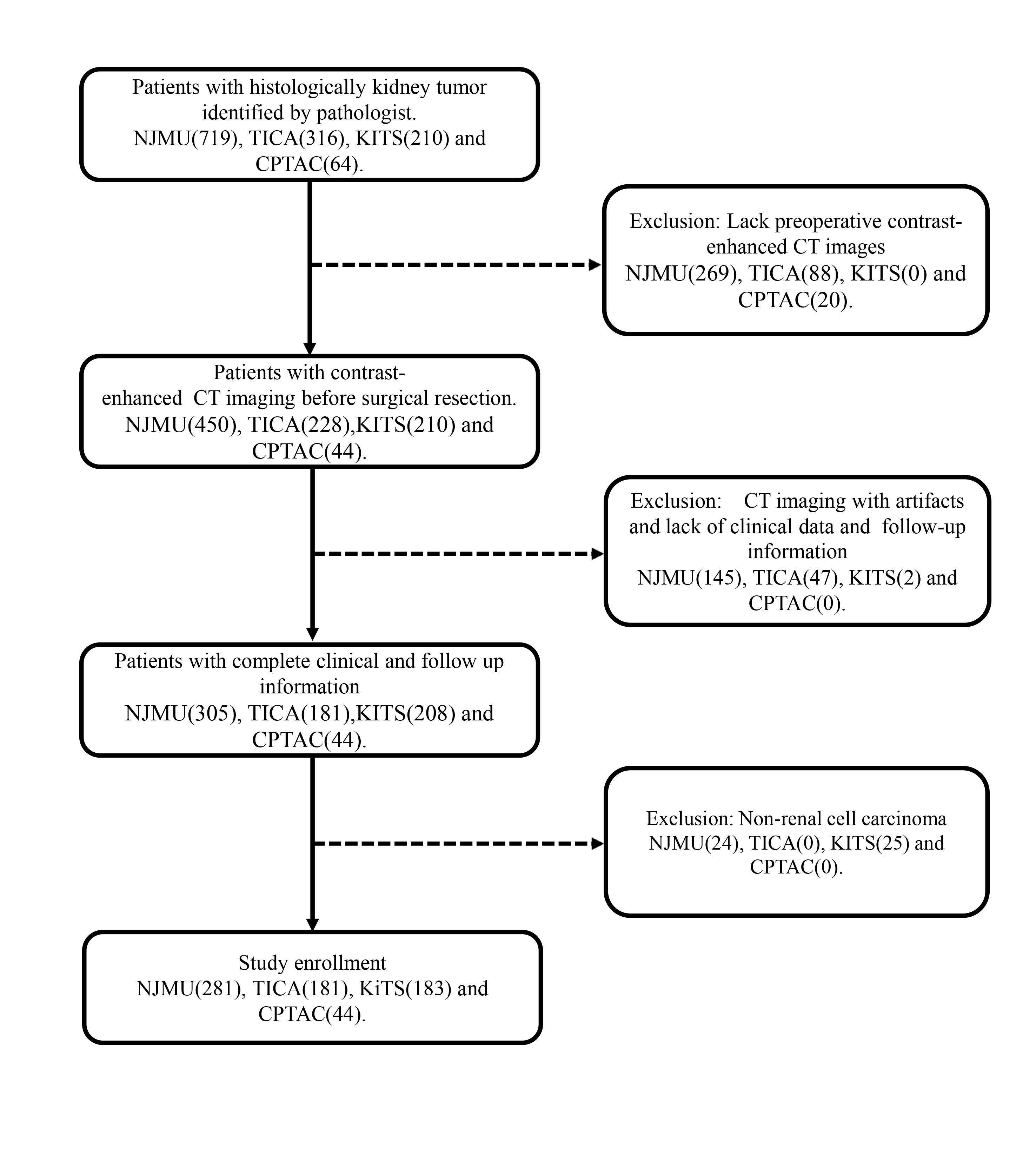

Supplement: Supplementary Figure 1 — Flow chart of the study population enrolment with inclusion and exclusion criteria. [file Image_1.tif]

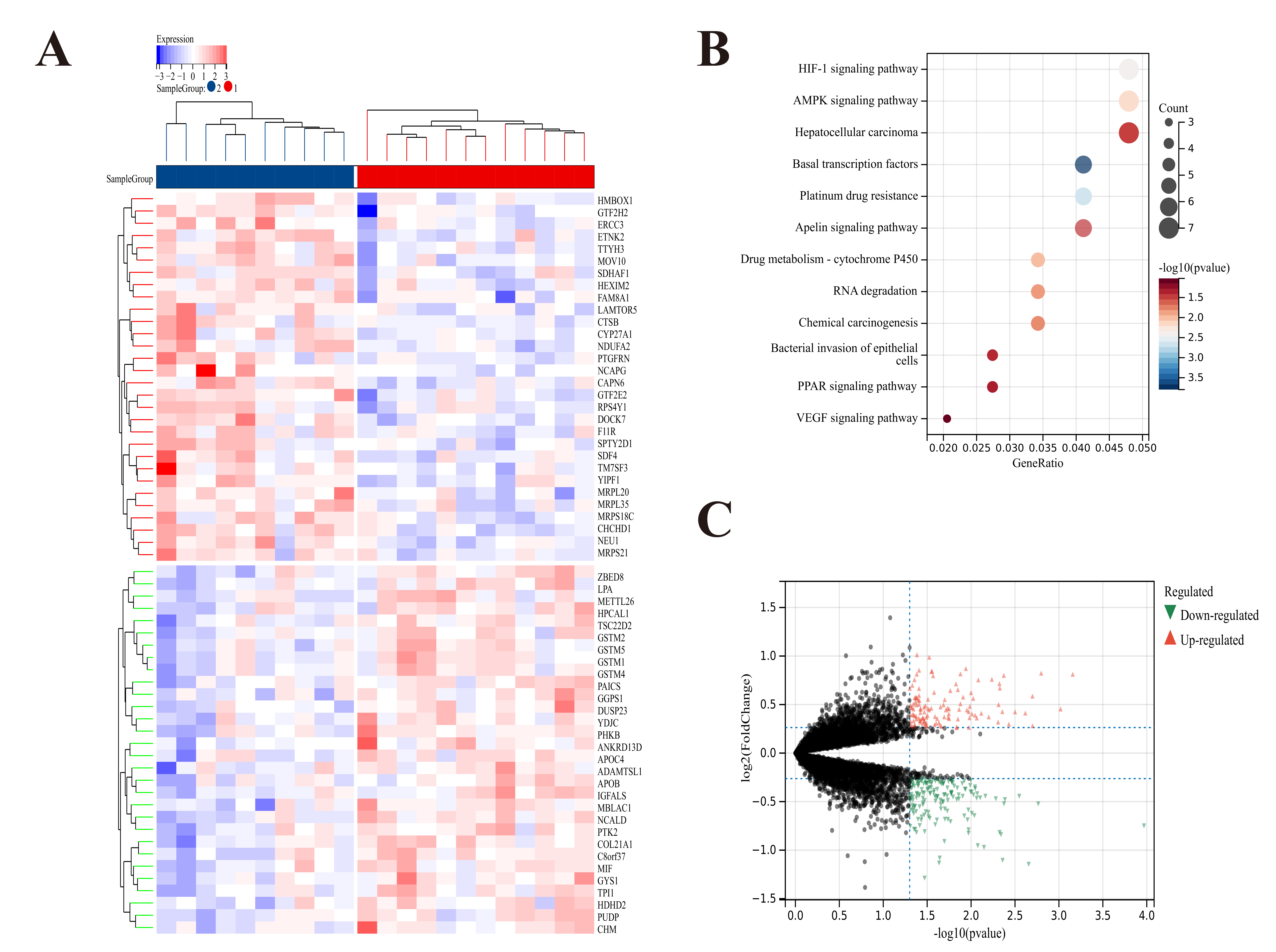

Supplement: Supplementary Figure 4 — The relationship between radiomics and genomics in CPTAC. (A) Hierarchical clustering of 30 DEGs of RCC. (B) KEGG pathways analysis of DEGs associated with radiomics signature. (C) A volcano plot generated using the data of differentially expressed genes (DEGs) between high-risk and low-risk groups from CPTAC. [file Image_4.tif]
