# Supplementary material for: Incremental value of radiomics with machine learning to the existing prognostic models for predicting outcome in renal cell carcinoma
Source: Front Oncol. 2023 Apr 28;13:1036734. doi: 10.3389/fonc.2023.1036734 (PMC10175776; doi:10.3389/fonc.2023.1036734)
Supplement: Supplementary file 6 [file Table_1.docx]

Supplementary Material

Table

**TableS1**. The detail parameters of computed tomography devices involved in this study.

|  | Philips iCT 256 | Somatom force CT A50A |
| --- | --- | --- |
| Tube voltage (kV) | 120 | 110 |
| Tube current (mA) | 75 | 94 |
| Pitch | 0.993 | 0.55 |
| Field of view (FOV) (mm) | 350 | 350 |
| Rotation time (s) | 0.5 | 0.28 |
| Slice thickness (mm) | 1.5 | 1.5 |

**TableS2**. Demographics and clinical characteristics in the training and validation cohorts.

|  | **Entire cohort（N=689)** | **Training cohort（N=281)** | **Validation cohort**  **(N=408)** | |
| --- | --- | --- | --- | --- |
| **Centre** | **Total(N=689)** | **NJMU(N=281)** | **NCI(N=225)** | **KiTS(N=183)** |
| Age (n, %) |  |  |  |  |
| <60 | 170(24.67%) | 79(11.47%) | 41(5.95%) | 50(7.26%) |
| >=60 | 519(75.33%) | 202(29.32%) | 184(26.71%) | 133(19.30%) |
| Gender |  |  |  |  |
| Male | 457(66.33%) | 199(28.88%) | 146(21.19%) | 112(16.26%) |
| Female | 232(33.67%) | 82(11.90%) | 79(11.47%) | 71(10.30%) |
| BMI level (n, %) |  |  |  |  |
| BMI≤24 | 156(29.77%) | 129(24.62%) | 8(1.53%) | 19(3.63%) |
| BMI>24 | 368(70.23%) | 152(29.01%) | 52(9.92%) | 164(31.30%) |
| Surgery type |  |  |  |  |
| robotic | 126(18.29%) | 22(7.83%) | - | 104(56.83%) |
| laparoscopic | 260(37.74%) | 234(83.27%) | - | 26(14.21%) |
| open | 78(11.32%) | 25(8.90%) | - | 53(28.96%) |
| Surgical approach |  |  |  |  |
| transperitoneal | 171(24.82%) | 22(7.83%) | - | 149(81.42%) |
| Retroperitoneal | 293(42.53%) | 259(92.17%) | - | 34(18.58%) |
| Tumor subtypes (n, %) |  |  |  |  |
| Clear cell RCC | 564(81.86%) | 219(31.79%) | 202(29.32%) | 143(20.75%) |
| Papillary RCC | 69(10.01%) | 31(4.50%) | 17(2.47%) | 21(3.05%) |
| Chromophobe RCC | 56(8.13%) | 31(4.50%) | 6(0.87%) | 19(2.76%) |
| Overall Survival (months) |  |  |  |  |
| Mean±SD | 41.74±26.10 | 53.78±23.79 | 34.43±23.45 | 32.24±25.44 |
| Status |  |  |  |  |
| Alive | 584(84.76%) | 251(36.43%) | 170(24.67%) | 163(23.66%) |
| Death | 105(15.24%) | 30(4.35%) | 55(7.98%) | 20(2.90%) |
| Laterality (n, %) |  |  |  |  |
| Left | 347(50.36%) | 149(21.63%) | 106(15.38%) | 92(13.35%) |
| Right | 342(49.64%) | 132(19.16%) | 119(17.27%) | 91(13.21%) |
| Location (n, %) |  |  |  |  |
| Upper | 212(30.77%) | 89(12.92%) | 70(10.16%) | 53(7.69%) |
| Interpole | 290(42.09%) | 108(15.67%) | 113(16.40%) | 69(10.01%) |
| Lower | 187(27.14%) | 84(12.19%) | 42(6.10%) | 61(8.85%) |
| Radiological diameter |  |  |  |  |
| Mean±SD | 5.25±2.97 | 4.92±2.67 | 6.02±3.11 | 4.82±3.05 |
| Pathological diameter |  |  |  |  |
| Mean±SD | 4.61±2.83 | 4.31±2.50 | 5.79±2.86 | 4.78±3.19 |
| 11-CSHA Score/11 |  |  |  |  |
| Mean±SD | - | 0.18±0.08 | - | 0.12±0.06 |
| WHO/ISUP (n, %) |  |  |  |  |
| 1 | 58(12.50%) | 31(6.68%) | - | 27(5.82%) |
| 2 | 269(57.97%) | 170(36.64%) | - | 99(21.34%) |
| 3 | 111(23.92%) | 68(14.66%) | - | 43(9.27%) |
| 4 | 26(5.60%) | 12(2.59%) | - | 14(3.02%) |
| SSIGN |  |  |  |  |
| Mean±SD | 2.29±2.66 | 2.04±2.61 | - | 2.67±2.69 |
| SSIGN Level |  |  |  |  |
| High Risk | 82(17.67%） | 51(18.15%) | - | 31(16.94%) |
| Intermediate Risk | 76(16.38%) | 30(10.68%) | - | 46(25.14%) |
| Low Risk | 306(65.95%) | 200(71.17%) | - | 106(57.92%) |
| Necrosis |  |  |  |  |
| No | 362(78.02%) | 220(47.41%) | - | 142(30.60%) |
| Yes | 102(21.98%) | 61(13.15%) | - | 41(8.84%) |
| Margin |  |  |  |  |
| Smooth | 365(52.98%) | 216(31.35%) | 74(10.74%) | 75(10.89%) |
| No Smooth | 324(47.02%) | 65(9.43%) | 151(21.92%) | 108(15.67%) |
| Growth Pattern |  |  |  |  |
| Endogenous | 69(10.01%) | 28(4.06%) | 8(1.16%) | 33(4.79%) |
| External | 620(89.99%) | 253(36.72%) | 217(31.49%) | 150(21.77%) |
| TNM Stage |  |  |  |  |
| I | 417(60.52%) | 193(28.01%) | 117(16.98%) | 107(15.53%) |
| II | 47(6.82%) | 14(2.03%) | 19(2.76%) | 14(2.03%) |
| III | 153(22.21%) | 58(8.42%) | 58(8.42%) | 37(5.37%) |
| IV | 72(10.45%) | 16(2.32%) | 31(4.50%) | 25(3.63%) |
| Pathological T stage |  |  |  |  |
| T1 | 442(64.15%) | 193(28.01%) | 121(17.56%) | 128(18.58%) |
| T2 | 51(7.402%) | 15(5.338%) | 26(11.56%) | 10(5.464%) |
| T3 | 174(25.25%) | 58(20.64%) | 73(32.44%) | 43(23.50%) |
| T4 | 14(2.03%) | 7(1.02%) | 5(0.73%) | 2(0.29%) |
| TX | 8(1.16%) | 8(1.16%) | - | - |
| Pathological N stage |  |  |  |  |
| N0 | 414(60.09%) | 274(39.77%) | 65(9.43%) | 75(10.89%) |
| N1 | 20(2.90%) | 6(0.87%) | 8(1.16%) | 6(0.87%) |
| NX | 255(37.01%) | 1(0.15%) | 152(22.06%) | 102(14.80%) |
| Pathological M stage |  |  |  |  |
| M0 | 496(71.99%) | 272(39.48%) | 145(21.04%) | 79(11.47%) |
| M1 | 48(6.97%) | 9(1.31%) | 23(3.34%) | 16(2.32%) |
| MX | 145(21.04%) | - | 57(8.27%) | 88(12.77%) |

**TableS3**. The top five percent of the 851variables, 43 radiomics features importance score from random forest models.

| Variable | Importance Score |
| --- | --- |
| wavelet_HLH_glszm_SmallAreaEmphasis | 1.821439845 |
| wavelet_HHL_glszm_SmallAreaLowGrayLevelEmphasis | 1.572590221 |
| wavelet_HHH_glszm_SizeZoneNonUniformityNormalized | 1.525135705 |
| wavelet_HHL_glszm_SmallAreaEmphasis | 1.503535922 |
| wavelet_HLH_glszm_SmallAreaLowGrayLevelEmphasis | 1.48808734 |
| wavelet_LHH_glszm_GrayLevelNonUniformityNormalized | 1.47613343 |
| wavelet_LHH_glszm_SmallAreaEmphasis | 1.227468741 |
| wavelet_LLH_glszm_SizeZoneNonUniformityNormalized | 1.194126221 |
| original_shape_Elongation | 1.192205789 |
| wavelet_HLL_ngtdm_Strength | 0.994708474 |
| wavelet_LHL_ngtdm_Strength | 0.896506944 |
| original_shape_Sphericity | 0.841123142 |
| wavelet_HHH_glcm_Imc2 | 0.832088179 |
| wavelet_HHH_firstorder_Skewness | 0.829568662 |
| wavelet_HHH_glszm_SmallAreaLowGrayLevelEmphasis | 0.822119973 |
| wavelet_LLL_firstorder_Maximum | 0.818689178 |
| wavelet_HLH_glcm_DifferenceVariance | 0.795567927 |
| wavelet_HHH_firstorder_Kurtosis | 0.77327278 |
| wavelet_HLL_glcm_ClusterProminence | 0.764165969 |
| wavelet_HHH_glcm_Imc1 | 0.759440354 |
| wavelet_HHL_ngtdm_Coarseness | 0.755762221 |
| wavelet_LLL_ngtdm_Strength | 0.753076056 |
| wavelet_HLH_ngtdm_Coarseness | 0.741287103 |
| wavelet_LHH_glszm_SmallAreaLowGrayLevelEmphasis | 0.718897427 |
| wavelet_HHH_glcm_MCC | 0.706852476 |
| wavelet_LLL_firstorder_Kurtosis | 0.705538862 |
| original_glszm_SmallAreaHighGrayLevelEmphasis | 0.702265588 |
| original_shape_Flatness | 0.694956245 |
| wavelet_LHH_ngtdm_Coarseness | 0.680867618 |
| wavelet_HLH_glcm_Correlation | 0.677582293 |
| wavelet_HHL_ngtdm_Strength | 0.674608401 |
| wavelet_HHL_glszm_LowGrayLevelZoneEmphasis | 0.671853248 |
| wavelet_HLL_ngtdm_Coarseness | 0.671252764 |
| original_firstorder_Maximum | 0.666776049 |
| wavelet_LLL_firstorder_Skewness | 0.662065149 |
| original_firstorder_Skewness | 0.655862462 |
| original_ngtdm_Coarseness | 0.64965854 |
| wavelet_LHL_firstorder_Variance | 0.643831831 |
| wavelet_LLL_ngtdm_Coarseness | 0.64177467 |
| wavelet_HHH_ngtdm_Coarseness | 0.639802392 |
| wavelet_HLH_ngtdm_Strength | 0.634071816 |
| wavelet_HHH_glrlm_LongRunHighGrayLevelEmphasis | 0.633443865 |
| wavelet_HLH_glszm_SizeZoneNonUniformityNormalized | 0.630626824 |

**TableS4**. Multivariate-COX regression coefficients for the 11 radiomics features selected by radiomics signature.

| **Variable** | **Coefficients** | **HR (95%CI)** | **P value** |
| --- | --- | --- | --- |
| wavelet_HHH_glcm_Imc2 | 0.0697 | 1.0722(0.7941,1.4476) | 0.6491 |
| wavelet_HHH_glrlm_LongRunHighGrayLevelEmphasis | -0.005 | 0.995(0.7309,1.3547) | 0.9749 |
| wavelet_HHH_glszm_SizeZoneNonUniformityNormalized | -0.1594 | 0.8527(0.703,1.0342) | 0.1056 |
| wavelet_HHH_ngtdm_Coarseness | -1.2316 | 0.2918(0.1044,0.8155) | 0.0188 |
| wavelet_HLH_glcm_DifferenceVariance | -0.2092 | 0.8112(0.4876,1.3495) | 0.4204 |
| wavelet_HLH_glszm_SmallAreaLowGrayLevelEmphasis | -0.0921 | 0.912(0.7304,1.1386) | 0.4159 |
| wavelet_LHH_glszm_GrayLevelNonUniformityNormalized | 0.3217 | 1.3794(1.0566,1.8009) | 0.0181 |
| wavelet_LHH_glszm_SmallAreaEmphasis | 0.1189 | 1.1263(0.89,1.4253) | 0.3223 |
| wavelet_LHL_firstorder_Variance | 0.081 | 1.0844(0.8451,1.3914) | 0.5242 |
| wavelet_LHL_ngtdm_Strength | -0.1189 | 0.8879(0.548,1.4385) | 0.629 |
| wavelet_LLH_glszm_SizeZoneNonUniformityNormalized | 0.3168 | 1.3727(1.0347,1.8211) | 0.028 |

**TableS5**. Multivariate and Univariable COX Analysis of Clinical prognostic factors in the training cohort.

| Variable | **Univariate analysis** | | | **Multivariable analysis** | | |
| --- | --- | --- | --- | --- | --- | --- |
|  | Beta | HR(95%CI) | P value | Beta | HR(95%CI) | P value |
| Age |  |  |  |  |  |  |
| <60 | Reference |  |  | Reference |  |  |
| >=60 | 0.869 | 2.385 (1.358-4.19) | 0.002 | 0.82 | 2.26(1.28,4.01) | 0.01 |
| Gender |  |  |  |  |  |  |
| Male | Reference |  |  | Reference |  |  |
| Female | 0.14 | 1.15 (0.7735-1.709) | 0.49 | 0.0029 | 1(0.67,1.5) | 0.99 |
| BMI level |  |  |  |  |  |  |
| BMI≤24 | Reference |  |  | Reference |  |  |
| BMI>24 | -0.208 | 0.8123 (0.5426-1.216) | 0.313 | -0.26 | 0.77(0.52,1.16) | 0.22 |
| Laterality |  |  |  |  |  |  |
| Left | Reference |  |  | Reference |  |  |
| Right | -0.335 | 0.7155 (0.4841-1.058) | 0.093 | -0.24 | 0.79(0.53,1.17) | 0.24 |
| Location |  |  |  |  |  |  |
| Upper | Reference |  |  | Reference |  |  |
| Interpole | 0.01 | 1.01(0.659,1.548) | 0.965 | 0.08 | 1.08(0.7,1.67) | 0.72 |
| Lower | -0.54 | 0.583(0.331,1.026) | 0.062 | -0.62 | 0.54(0.3,0.95) | 0.03 |
| Radiological diameter | 0.149 | 1.161 (1.104-1.221) | <0.001 | 0.14 | 1.15(1.09,1.21) | <0.001 |
| Margin |  |  |  |  |  |  |
| Smooth | Reference |  |  | Reference |  |  |
| No Smooth | 0.401 | 1.494 (1.016-2.195) | 0.041 | 0.12 | 1.13(0.76,1.69) | 0.55 |
| Growth Pattern |  |  |  |  |  |  |
| Endogenous | Reference |  |  | Reference |  |  |
| External | 1.274 | 3.573 (1.131-11.29) | 0.03 | 0.72 | 2.06(0.63,6.71) | 0.23 |

**TableS6**. Multivariate-COX regression coefficients for the variables selected by clinical nomogram.

| Variable | Beta | HR (95%CI) | P value |
| --- | --- | --- | --- |
| Age |  |  |  |
| <60 | Reference |  |  |
| >=60 | 0.885 | 2.423(1.375,4.27) | 0.002 |
| Location |  |  |  |
| Upper | Reference |  |  |
| Interpole | 0.08 | 1.083(0.705,1.664) | 0.715 |
| Lower | -0.604 | 0.547(0.31,0.965) | 0.037 |
| Radiological diameter | 0.154 | 1.166(1.107,1.228) | <0.001 |

**TableS7**. Multivariate-COX regression coefficients for the variables selected by radiomics nomogram.

| Variable | Beta | HR (95%CI) | P value |
| --- | --- | --- | --- |
| Radiomics-Signature | 0.788 | 2.2(1.794,2.697) | <0.001 |
| Clinical-Score | 0.258 | 1.294(0.896,1.868) | 0.169 |
| SSIGN | -0.086 | 0.918(0.816,1.033) | 0.154 |
| WHOISUP |  |  |  |
| WHOISUP1 | Reference |  |  |
| WHOISUP2 | 0.292 | 1.339(0.398,4.507) | 0.638 |
| WHOISUP3 | 1.528 | 4.611(1.349,15.765) | 0.015 |
| WHOISUP4 | 2.292 | 9.895(2.663,36.769) | 0.001 |
| TNM Stage |  |  |  |
| Stage I | Reference |  |  |
| Stage II | -1.265 | 0.282(0.098,0.814) | 0.019 |
| Stage III | 0.12 | 1.127(0.55,2.31) | 0.744 |
| Stage IV | 0.685 | 1.984(0.944,4.169) | 0.071 |

**TableS8.** The Bland-Altman analysis for 11 radiomics features.

| Radiomics Features | Mean  (Observer1) | Mean  (Observer2) | Mean  (Difference) | 95% CI  (Difference) |
| --- | --- | --- | --- | --- |
| wavelet_HHH_glcm_Imc2 | 0.17 | 0.14 | 0.03 | -0.078 ~ 0.137 |
| wavelet_HHH_glrlm_LongRunHighGrayLevelEmphasis | 19.65 | 19.29 | 0.37 | -15.403 ~ 16.134 |
| wavelet_HHH_glszm_SizeZoneNonUniformityNormalized | 0.38 | 0.43 | -0.05 | -0.415 ~ 0.316 |
| wavelet_HHH_ngtdm_Coarseness | 0.00 | 0.00 | 0.00 | -0.005 ~ 0.010 |
| wavelet_HLH_glcm_DifferenceVariance | 0.31 | 0.28 | 0.03 | -0.087 ~ 0.138 |
| wavelet_HLH_glszm_SmallAreaLowGrayLevelEmphasis | 0.17 | 0.24 | -0.07 | -0.388 ~ 0.242 |
| wavelet_LHH_glszm_GrayLevelNonUniformityNormalized | 0.33 | 0.40 | -0.07 | -0.315 ~ 0.174 |
| wavelet_LHH_glszm_SmallAreaEmphasis | 0.49 | 0.50 | -0.01 | -0.278 ~ 0.254 |
| wavelet_LHL_firstorder_Variance | 645.32 | 684.30 | -38.98 | -501.515 ~ 423.553 |
| wavelet_LHL_ngtdm_Strength | 0.08 | 0.05 | 0.03 | -0.128 ~ 0.185 |
| wavelet_LLH_glszm_SizeZoneNonUniformityNormalized | 0.20 | 0.18 | 0.02 | -0.076 ~ 0.114 |
